# Unsupervised Domain Adaptation Method Based on Relative Entropy Regularization and Measure Propagation

**DOI:** 10.3390/e27040426

**Published:** 2025-04-14

**Authors:** Lianghao Tan, Zhuo Peng, Yongjia Song, Xiaoyi Liu, Huangqi Jiang, Shubing Liu, Weixi Wu, Zhiyuan Xiang

**Affiliations:** 1 Department of Computer Science, Arizona State University, Tempe, AZ 85281, USA; zhuo560912@gmail.com (Z.P.); xliu472@asu.edu (X.L.); 2 Department of Language Science, University of California, Irvine, CA 92697, USA; yongjias523@gmail.com; 3 Department of Computer Science, Georgia Institute of Technology, Atlanta, GA 30332, USA; 4 Department of Computer Science, North Carolina at Chapel Hill, Orange, GA 27599, USA; sliu2@unc.edu; 5 Department of Computer Science, New York University, Brooklyn, NY 10003, USA; ww2147@nyu.edu; 6 Department of Computer Science, University of California, San Diego, CA 92093, USA; z1xiang@uced.edu

**Keywords:** unsupervised domain adaptation, information theory, relative entropy regularization, probability measure

## Abstract

This paper presents a novel unsupervised domain adaptation (UDA) framework that integrates information-theoretic principles to mitigate distributional discrepancies between source and target domains. The proposed method incorporates two key components: (1) relative entropy regularization, which leverages Kullback–Leibler (KL) divergence to align the predicted label distribution of the target domain with a reference distribution derived from the source domain, thereby reducing prediction uncertainty; and (2) measure propagation, a technique that transfers probability mass from the source domain to generate pseudo-measures—estimated probabilistic representations—for the unlabeled target domain. This dual mechanism enhances both global feature alignment and semantic consistency across domains. Extensive experiments on benchmark datasets (OfficeHome and DomainNet) demonstrate that the proposed approach consistently outperforms State-of-the-Art methods, particularly in scenarios with significant domain shifts. These results confirm the robustness, scalability, and theoretical grounding of our framework, offering a new perspective on the fusion of information theory and domain adaptation.

## 1. Introduction

Unsupervised domain adaptation (UDA) is an important research direction in the field of machine learning, aimed at addressing the problem of distributional differences between source and target domains [1]. In many real-world applications, such as image classification, speech recognition, and natural language processing, acquiring labeled data is costly, and the target domain often lacks labels [2,3]. Furthermore, data from the source domain cannot be directly used for the target task. UDA enhances the performance of models in the target domain by leveraging labeled information from the source domain, which has broad practical applications [4]. For example, in medical image analysis, UDA can help adapt data across hospitals. In autonomous driving, UDA can adapt to different lighting conditions or viewpoints. Therefore, the study of efficient UDA methods is of significant theoretical and practical importance in advancing cross-domain generalization capabilities.

Although traditional unsupervised domain adaptation (UDA) methods, such as feature alignment and adversarial training, have alleviated distribution discrepancies to some extent, they still face notable limitations. A key challenge lies in the uncertainty of the predicted distribution in the target domain, which often leads to overfitting or reduced accuracy. Moreover, many existing approaches rely on shallow metrics, such as Maximum Mean Discrepancy (MMD) or adversarial loss, to align source and target domains, without incorporating deeper insights from information theory. As a result, these methods may overlook the intrinsic structural relationships between domains, causing critical information to be lost or redundant features to be introduced, ultimately impairing generalization capability in complex scenarios [5].

To address these limitations, there is a growing interest in exploring more principled approaches that can provide deeper theoretical guarantees while capturing both global and semantic structures in cross-domain tasks. Information theory, with its ability to model uncertainty and quantify distributional differences, offers a compelling foundation for designing such methods. However, its integration into domain adaptation frameworks remains underexplored.

In light of this, we propose a novel information-theoretic framework for UDA that explicitly addresses distribution discrepancy, defined as the difference in data distributions between the labeled source domain and the unlabeled target domain. Our method integrates relative entropy regularization and measure propagation to achieve robust domain alignment from an information-theoretic perspective. Specifically, relative entropy regularization employs Kullback–Leibler (KL) divergence to constrain the target domain’s predicted distribution, encouraging consistency with the source domain’s reference distribution and reducing information loss. Meanwhile, measure propagation transfers probability measures from the source to the target domain, constructing pseudo-measures that ensure global consistency in feature space representation [6]. These two components are jointly optimized with a feature extractor and classifier, resulting in improved performance and generalization across diverse target domains [7,8].

The innovations of this paper are summarized as follows:

(1) An unsupervised domain adaptation method based on relative entropy regularization is proposed. KL divergence is used to accurately constrain the distributional difference between the source domain and the target domain from the perspective of information theory, thus overcoming the limitations of traditional distance metrics.
(2) The measure propagation mechanism is introduced, generating the target domain pseudo-measure by propagating the probability measure of the source domain, and the application of information theory in the global structure modeling of the feature space is deepened;
(3) Combining the above methods, we construct an information-theory-driven joint optimization framework, which significantly improves the generalization ability of the model in the target domain and provides new ideas for the application of information theory in deep learning.

## 2. Related Work

### 2.1. Unsupervised Domain Adaptation

Unsupervised domain adaptation (UDA) aims to address the issue of distribution discrepancies between source and target domains, which is critical in fields like cross-domain image classification and semantic segmentation. Due to the lack of labeled data in the target domain, UDA leverages supervised information from the source domain and unsupervised data from the target domain to train models that generalize well in the target domain [9]. Traditional methods often reduce the distribution gap through feature alignment or adversarial training. In recent years, self-supervised learning and pseudo-labeling techniques have been widely explored in UDA. However, existing methods still face limitations when dealing with out-of-distribution samples or global structure alignment, providing room for further improvement [10].

Unsupervised domain adaptation has gained significant attention from both academia and industry in recent years, becoming one of the key technologies in cross-domain knowledge transfer. For instance, Xu et al. [11] proposed a Transformer-based UDA method, CDTrans, which learns source/target features and aligns domains using a shared-weight three-branch Transformer framework. To generate accurate pseudo-labels, the paper also introduced a bidirectional center-aware labeling algorithm, optimizing the pseudo-label generation process for target domain samples. Mirza et al. [12] introduced a dynamic unsupervised adaptation method, DUA, which continuously adapts the statistics of the batch normalization layer to adjust feature representations. This approach achieves significant performance improvements with minimal computational overhead, making it suitable for any network architecture using batch normalization. Huang et al. [13] proposed a novel category contrast technique, CaCo, which introduces semantic priors into instance contrast learning to improve UDA performance. CaCo constructs a semantic-aware dictionary and assigns pseudo-category labels to target domain samples based on source domain category priors, facilitating category-discriminative and domain-invariant feature representations.

In conclusion, this paper provides an overview of the recent advancements in unsupervised domain adaptation (UDA), with a focus on methods tackling challenges such as the lack of labeled data in the target domain, feature alignment, and category discrimination. While existing approaches, such as adversarial learning and self-supervised strategies, have demonstrated effectiveness, they often fall short in modeling distributional uncertainty and preserving global structural information. In contrast, the proposed method introduces relative entropy regularization and measure propagation, which directly address these limitations by leveraging information-theoretic principles. Relative entropy regularization constrains the predictive uncertainty in the target domain, while measure propagation facilitates global feature alignment through probability-based modeling. These innovations fill critical gaps in current UDA techniques, offering a theoretically grounded and practically effective solution for improving cross-domain generalization across diverse tasks.

### 2.2. Information Theory in Deep Learning

In recent years, the application of information theory in deep learning has garnered significant attention, especially in understanding and optimizing deep neural networks [14,15]. Classical concepts of information theory, such as mutual information, KL divergence, and entropy, have been incorporated into the training processes of deep learning models to design more effective loss functions and regularization methods [16,17]. For instance, the information bottleneck method optimizes the flow of information within deep networks by minimizing the loss of information between the input and output, thereby improving the model’s generalization ability [18,19]. Moreover, information theory has been applied to network structure design and adjustments by quantifying information transfer and compression within networks, further enhancing model efficiency and expressive power.

At the same time, the application of information theory has achieved notable progress in fields such as unsupervised learning and Generative Adversarial Networks (GANs). In unsupervised learning, information theory is used for analyzing the stability of adversarial learning and structuring the latent space, particularly in Variational Autoencoders (VAEs). Here, KL divergence is widely used as part of the loss function to optimize the distribution of the latent representation, enabling the generative model to approximate the true data distribution effectively. For example, Wu et al. [20] proposed a robust dynamic semi-supervised symmetric regularization image blur clustering algorithm, which incorporates KL divergence and spatial information constraints. By introducing weighted squared Euclidean distance and maximum entropy fuzzy clustering, this method improves the robustness and performance of image segmentation in high-noise environments. Sanokowski et al. [21] proposed a new method that uses an upper bound of the reverse Kullback–Leibler divergence loss function. This method breaks through the traditional generative model’s requirement for exact sample likelihoods, enabling the use of highly expressive latent variable models like diffusion models. The method does not require training data and allows for data-independent sample learning in areas like combinatorial optimization.

In the context of GANs, information-theory methods help clarify the adversarial game between the generator and discriminator. By introducing information gain, these methods improve the generation ability and stability of the model. Lee et al. [22] introduced a new framework that simultaneously alleviates catastrophic forgetting in the discriminator and mode collapse in the generator through contrastive learning and mutual information maximization in adversarial learning. Li et al. [23] applied information theory and GANs to recommendation algorithms, proposing a fairness-aware learning algorithm, FairGAN. FairGAN maps exposure fairness issues to negative bias in implicit feedback data. It dynamically generates fairness signals using a novel fairness-aware learning strategy, optimizing ranking search direction while ensuring fairness in exposure distribution across items, without compromising user utility.

## 3. Method

**Figure 1:**
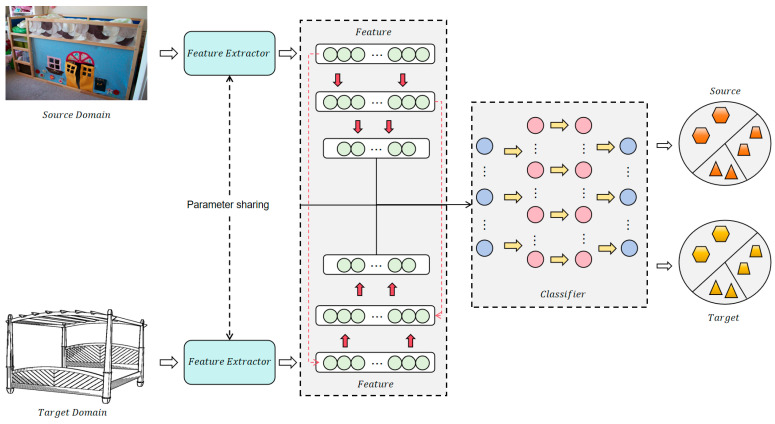
The network architecture used in this article.

In this study, we propose a novel unsupervised domain adaptation method that addresses the distributional gap between a labeled source domain and an unlabeled target domain by integrating two complementary information-theoretic components: relative entropy regularization and measure propagation. The model architecture is shown in Figure 1.

The first component, relative entropy regularization, is formulated using Kullback–Leibler (KL) divergence to minimize the discrepancy between the predicted label distribution in the target domain and a reference distribution constructed from the source domain. This regularization encourages the model to generate predictions in the target domain that are statistically consistent with the knowledge obtained from the labeled source domain, thereby reducing uncertainty and enhancing model robustness.

To further address the structural misalignment in the feature space, we incorporate a second component: measure propagation. This mechanism aims to build an estimated distribution, or pseudo-measure, for the target domain by transferring probability information from the source domain. Specifically, the probability distribution of source features is adjusted using a learned transformation function to approximate the distribution of target features. This transformation is modeled as a density ratio, which quantifies how the source distribution should be reshaped to resemble the target one. The density ratio is implemented via a neural network that outputs a scaling factor for each target feature, enabling flexible and data-driven alignment.

The optimization process is composed of three loss functions: (1) a classification loss on the labeled source data, (2) a KL divergence loss enforcing consistency between the predicted target distribution and the source-derived reference distribution, and (3) an adversarial loss that promotes similarity between the propagated pseudo-measure and the actual distribution of the target domain. These components are jointly optimized during training to update the feature extractor, classifier, and auxiliary networks. This unified framework results in more stable cross-domain generalization and improved alignment at both the distributional and structural levels [24,25].

### 3.1. Information-Theoretic Constraint via Relative Entropy Regularization

In unsupervised domain adaptation (UDA), the distributional difference between the source domain $D_{s}={\left\{(x_{s}^{i},y_{s}^{i})\right\}}_{i=1}^{N}$ and target domain $D_{t}={\left\{(x_{t}^{i})\right\}}_{i=1}^{N}$ $P_{s}=(x,y)\neq P_{t}(x,y)$ is a central challenge. Traditional methods usually solve the problem through feature alignment or adversarial training, but these methods struggle to accurately control the prediction behavior of the target domain due to the lack of labels in the target domain. Therefore, we propose a regularization strategy based on relative entropy (i.e., KL divergence) to achieve domain adaptation by restricting the consistency between the target domain distribution and the source-domain-derived reference distribution.

**Figure 2:**
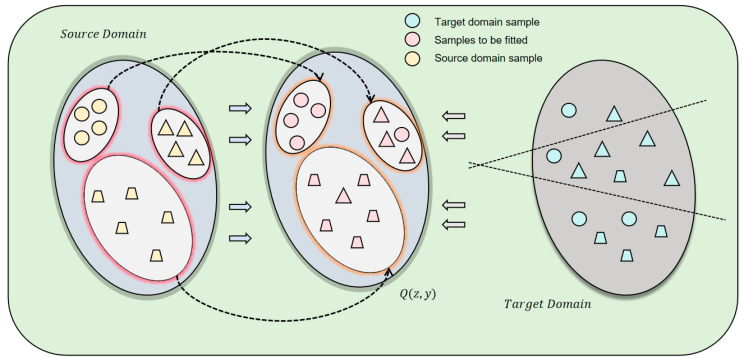
This figure shows the network architecture used in the article, showing the relationship between source and target domain samples. By introducing a reference distribution Q(z,y) and regularizing the joint distribution $P_{t}(z,y)$ of the target domain, the network aims to transfer source domain knowledge to the target domain.

We define a feature extractor G=X→Z with parameters $\theta_{G}$, which maps input xϵX to feature space Z and obtains z=G(x). The classifier $C:Z\to {∆}^{K}$ with parameters $\theta_{C}$ takes input z and outputs the probability distribution K of P(y|z)=C(z) categories. The source domain has supervised data, and the feature distribution $P_{Z_{s}}(z)$ and conditional distribution $P_{S}(y|z)$ can be directly defined. For the target domain, only $P_{Z_{t}}(z)$ can be obtained through $G(x_{t})$, and $P_{t}(y|z)$ is unknown because it has no labels. Our goals are as follows: (1) to train G and C on the source domain to minimize the classification error; (2) to ensure that the joint distribution $P_{t}(z,y)=P_{Z_{t}}(z)P_{t}(y|z)$ of the target domain is aligned with the source domain knowledge. To this end, we introduce a reference distribution Q(z,y) and regularize $P_{t}(z,y)$ with relative entropy to make it close to Q(z,y). The model architecture of this section is shown in Figure 2.

The reference distribution Q(z,y) is constructed directly from the source domain and is defined as follows:

(1) $$Q(z,y)=P_{Z_{s}}(z)P_{s}(y|z)$$

where $P_{Z_{s}}(z)$ is the empirical distribution of source domain features.

(2) $$P_{Z_{s}}(z)=\frac{1}{N_{s}}\sum_{i=1}^{N_{s}}\delta (z-G(x_{s}^{i}))$$

δ is the Dirac delta function, and $P_{S}(y|z)=C(G(x_{s}))$ is the classifier’s prediction of the source domain features. This form is the simplest and most direct choice, making full use of the empirical distribution of the source domain and the classifier output to avoid introducing additional complexity. The joint distribution of the target domain is as follows:

(3) $$P_{t}(z,y)=P_{Z_{t}}(z)P_{t}(y|z)\;\;\;$$

where $P_{Z_{t}}(z)=\frac{1}{N_{t}}\sum_{j=1}^{N_{t}}\delta (z-G(x_{t}^{i})),\;P_{t}(y|z)=C(G(x_{t}))$. We want to minimize the relative entropy between $P_{t}(z,y)$ and Q(z,y):

(4) $$L_{re}=D_{KL}(P_{t}(z,y)||Q(z,y))\;\;$$

expands to:

(5) $$L_{re}=\int P_{t}(z,y)log\frac{P_{t}(z,y)}{Q(z,y)}dzdy\;\;$$

Substituting into the joint distribution, we can see the following:

(6) $$L_{re}=\int P_{Z_{t}}(z)P_{t}(y|z)log\frac{P_{Z_{t}}(z)P_{t}(y|z)}{P_{Z_{s}}(z)P_{s}(y|z)}dzdy\;\;$$

According to the properties of logarithms and the expected decomposition, we can obtain the following:

(7) $$L_{re}=\int P_{Z_{t}}(z)log\frac{P_{Z_{t}}(z)}{P_{Z_{s}}(z)}dz+\int P_{Z_{t}}(z)[\int P_{t}(y|z)log\frac{P_{t}(y|z)}{P_{s}(y|z)}dy]dz$$

which can be equivalently changed to the following:

(8) $$L_{re}=D_{KL}(P_{Z_{t}}(z)||P_{Z_{s}}(z))+E_{z\sim P_{Z_{t}}}[D_{KL}(P_{t}(y|z)||P_{s}(y|z))]\;\;$$

The first term $D_{KL}(P_{Z_{t}}(z)||P_{Z_{s}}(z))$ measures the difference in feature distribution, and the second term $E_{z\sim P_{Z_{t}}}[D_{KL}(P_{t}(y|z)||P_{s}(y|z))]$ ensures that the conditional distribution is consistent. Since it is not feasible to directly calculate the continuous integral, we use empirical samples to approximate. The first term is as follows:

(9) $$D_{KL}(P_{Z_{t}}(z)||P_{Z_{s}}(z))\approx \frac{1}{N_{t}}\sum_{j=1}^{N_{t}}log\frac{P_{Z_{t}}(G(x_{t}^{j}))}{P_{Z_{s}}(G(x_{t}^{j}))}\;$$

However, accurate estimation of $P_{Z_{s}}(z)$ and $P_{Z_{t}}(z)$ requires density estimation, which increases the computational burden. For simplicity, we assume that G has been aligned with $P_{Z_{s}}$ and $P_{Z_{t}}$ through subsequent methods (see Section 3.2), making the first term less influential. Therefore, we focus on optimizing the second term:

(10) $$L_{re}\approx E_{z\sim P_{Z_{t}}}[D_{KL}(P_{t}(y|z)||P_{s}(y|z))]$$

The empirical approximation is as follows:

(11) $$L_{re}\approx \frac{1}{N_{t}}\sum_{j=1}^{N_{t}}D_{KL}(C(G(x_{t}^{j}))||P_{s}(y|G(x_{t}^{j})))\;$$

Since the target domain sample $G(x_{t}^{j})$ has no corresponding $P_{s}(y|G(x_{t}^{j}))$, we use the source domain nearest neighbor approximation: for each $x_{t}^{j}$, find the nearest source domain sample $x_{s}^{k(j)}={argmin}_{x_{s}^{i}}{\left||G(x_{t}^{j})-G(x_{t}^{i})|\right|}_{2}$ and define:

(12) $$P_{s}(y|G(x_{t}^{j}))\approx C(G(x_{s}^{k(j)}))\;$$

So, we can obtain the following:

(13) $$L_{re}\approx \frac{1}{N_{t}}\sum_{j=1}^{N_{t}}\sum_{k=1}^{K}{C(G(x_{t}^{j}))}_{k}log\frac{{C(G(x_{t}^{j}))}_{k}}{{C(G(x_{t}^{k(j)}))}_{k}}\;$$

In summary, relative entropy regularization introduces KL divergence as an information-theory tool to constrain the consistency of the predicted distribution of the target domain and the reference distribution of the source domain, effectively reducing the distributional difference problem in unsupervised domain adaptation [25]. This method uses the supervised information of the source domain and the unlabeled data of the target domain, and through nearest neighbor approximation and empirical sample optimization, it significantly improves the robustness and prediction accuracy of the model in the target domain, providing a solid foundation for information-theory-driven domain adaptation.

### 3.2. Measure Propagation

**Figure 3:**
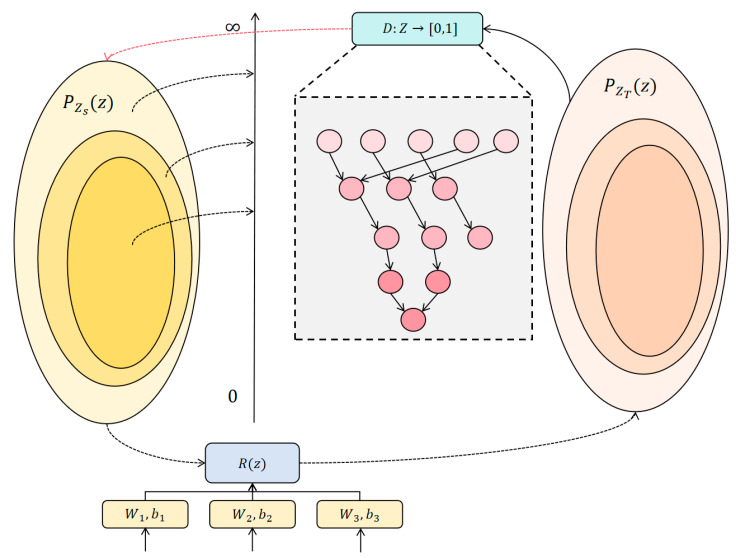
The figure mainly describes the knowledge transfer between the target domain and the source domain, which includes the propagation of the target domain measurement and the source domain measurement. Function R(z) is responsible for calculating the mapping between different domains, and the network is trained and adjusted through W and b.

In Section 3.2 we discussed that the goal of measure propagation is to derive the pseudo measure ${\tilde{P}}_{Z_{t}}(z)$ of the target domain from $P_{Z_{s}}(z)$, and make ${\tilde{P}}_{Z_{t}}(z)$ close to $P_{Z_{s}}(z)$ by optimizing G, thereby achieving knowledge transfer between domains. The model architecture is shown in Figure 3.

The core idea of measure propagation is to use the probability measure of the source domain and propagate it to the target domain through some transformation to generate a reference distribution consistent with the target domain samples. We define a measure propagator T:P(Z)×Z→P(Z), which inputs the source domain measure $P_{Z_{s}}(z)$ and the target domain feature $Z_{t}={\left\{G(x_{t}^{j})\right\}}_{j=1}^{N_{t}}$ and outputs a pseudo measure:

(14) $${\tilde{P}}_{Z_{t}}(z)=T(P_{Z_{s}}(z),Z_{t})\;$$

To simplify the implementation, we choose the simplest form based on density ratio. Assume that the target domain distribution $P_{Z_{t}}(z)$ is associated with the source domain distribution $P_{Z_{s}}(z)$ through a density ratio function r(z):

(15) $$P_{Z_{t}}(z)=r(z)P_{Z_{s}}(z)\;$$

Then, the pseudo-measure can be defined as follows:

(16) $${\tilde{P}}_{Z_{t}}(z)=r(z)P_{Z_{s}}(z)\;$$

where r(z) represents the distribution change ratio from the source domain to the target domain. There are many possible forms of direct estimation of r(z), such as parametric models or non-parametric estimation. To keep it simple, we chose a neural network $R:Z\to R^{+}$ with parameters $\theta_{R}$ to directly predict r(z)=R(z) and regard it as a scalar function of z. The source domain feature distribution is the empirical distribution:

(17) $$P_{Z_{s}}(z)=\frac{1}{N_{s}}\sum_{i=1}^{N_{s}}\delta (z-G(x_{s}^{i}))\;$$

So, the pseudo-measure is as follows:

(18) $${\tilde{P}}_{Z_{t}}(z)=R(z)\cdot \frac{1}{N_{t}}\sum_{i=1}^{N_{t}}\delta (z-G(x_{s}^{i}))\;$$

The actual feature distribution of the target domain is as follows:

(19) $$P_{Z_{t}}(z)=\frac{1}{N_{t}}\sum_{i=1}^{N_{t}}\delta (z-G(x_{t}^{j}))\;$$

Our goal is to make $P_{Z_{t}}(z)$ as close to ${\tilde{P}}_{Z_{t}}(z)$ as possible by optimizing *G* and *R*. The optimization goal uses KL divergence to measure the difference between the two:

(20) $$L_{mp}=D_{KL}(P_{Z_{t}}(z)||{\tilde{P}}_{Z_{t}}(z))$$

Expanding and inserting the definition provides the following:

(21) $$L_{mp}=\int P_{Z_{t}}(z)log\frac{P_{Z_{t}}(z)}{R(z)P_{Z_{s}}(z)}dz$$

Then, split the formula into three terms to obtain the following:

(22) $$L_{mp}=\int P_{Z_{t}}(z)logP_{Z_{t}}(z)dz-\int P_{Z_{t}}(z)logR(z)dz-\int P_{Z_{t}}(z)logP_{Z_{s}}(z)dz$$

The first term is the entropy of $P_{Z_{t}}(z)$, which has no direct contribution to optimization and can be ignored. The second and third terms are as follows:

(23) $$L_{mp}=-E_{z\sim P_{Z_{t}}}[logR(z)]-E_{z\sim P_{Z_{t}}}[logP_{Z_{s}}(z)]$$

The empirical approximation is as follows:

(24) $$L_{mp}\approx -\frac{1}{N_{t}}\sum_{j=1}^{N_{t}}logR(G(x_{t}^{j}))-\frac{1}{N_{t}}\sum_{j=1}^{N_{t}}log(\frac{1}{N_{s}}\sum_{i=1}^{N_{s}}\delta (G(x_{t}^{j})-G(x_{s}^{i})))$$

Since the δ function is zero at $G(x_{t}^{j})\neq G(x_{s}^{i})$, the third term is difficult to calculate directly. And Section 3.1 has been aligned, so this article focuses on optimizing R(z) and *G*:

(25) $$L_{mp}\approx -\frac{1}{N_{t}}\sum_{j=1}^{N_{t}}logR(G(x_{t}^{j}))$$

But this alone is not enough to constrain R(z). We introduce a discriminator D:Z→[0,1] with parameter $\theta_{D}$ to distinguish $z\sim P_{Z}$ and $z\sim {\tilde{P}}_{Z}$. The optimization goal is as follows:

(26) $$L_{D}=E_{z\sim P_{Z_{s}}}[logD(z)]+E_{z\sim {\tilde{P}}_{Z_{s}}}[log(1-D(z))]$$

The empirical form is as follows:

(27) $$L_{mp}=\frac{1}{N_{s}}\sum_{i=1}^{N_{s}}log(D(G(x_{s}^{i}))+\frac{1}{N_{t}}\sum_{j=1}^{N_{t}}log(1-D(G(x_{t}^{j})))R(G(x_{t}^{j}))$$

The goal of *G* and *R* is to deceive *D*, that is, to minimize the following:

(28) $$L_{mp}=\frac{1}{N_{s}}\sum_{i=1}^{N_{s}}log(1-D(G(x_{t}^{j})))R(G(x_{t}^{j}))$$

Measure propagation generates pseudo-measures of the target domain by propagating the probability measure of the source domain, achieving global alignment of the feature space and enhancing the distribution consistency of unsupervised domain adaptation. This method uses a simple form of density ratio and adversarial training to optimize the feature extractor and discriminator, significantly improving the generalization ability of the model in complex cross-domain tasks and deepening the application of information theory in global structure modeling.

### 3.3. Overall Loss Function

In our unsupervised domain adaptation framework, the overall loss function combines multiple information-theory-driven components to optimize the distribution alignment of the source and target domains. The core part includes the cross-entropy loss $L_{cls}$ of the source domain, which is used to minimize the classification error and is defined as follows:

(29) $$L_{cls}=\frac{1}{N_{s}}\sum_{i=1}^{N_{s}}{-logC(G(x_{s}^{i}))}_{y_{s}^{i}}$$

where $N_{s}$ is the number of source domain samples, $x_{s}^{i}$ and $y_{s}^{i}$ are source domain samples and labels, respectively, and $C(G(x_{s}^{i}))$ is the classifier prediction probability. Combined with the relative entropy regularization loss $L_{re}$, the consistency between the target domain prediction distribution and the source domain reference distribution is constrained by the KL divergence, and its empirical approximation is as follows:

(30) $$L_{re}\approx \frac{1}{N_{t}}\sum_{j=1}^{N_{t}}\sum_{k=1}^{K}{C(G(x_{t}^{j}))}_{k}log\frac{{C(G(x_{t}^{j}))}_{k}}{{C(G(x_{t}^{k(j)}))}_{k}}\;$$

where $N_{t}$ is the number of target domain samples, $x_{t}^{j}$ is the target domain sample, and $x_{t}^{k(j)}$ is the nearest neighbor source domain sample. In addition, the measure propagation loss $L_{mp}$ optimizes the alignment of the target domain pseudo measure with the source domain measure through adversarial training, which is defined as follows:

(31) $$L_{mp}=\frac{1}{N_{s}}\sum_{i=1}^{N_{s}}log(1-D(G(x_{t}^{j})))R(G(x_{t}^{j}))$$

where *D* is the discriminator and *R* is the density ratio network. The final overall loss function is as follows:

(32) $$L=L_{cls}+\lambda_{re}L_{re}+\lambda_{mp}L_{mp}$$

By balancing the contributions of the three through hyperparameters $\lambda_{re}$ and $\lambda_{mp}$, the feature extractor *G* and classifier *C* are jointly optimized to achieve information-theory-driven cross-domain generalization.

## 4. Datasets and Evaluation Metrics

### 4.1. Dataset Introduction

In this section, we provide an overview of the datasets used in this study. Specifically, two datasets are utilized: OfficeHome and DomainNet. These datasets are widely recognized in the domain adaptation research community and serve as benchmarks for evaluating various algorithms in the context of unsupervised domain adaptation. The OfficeHome dataset consists of a collection of images from different office-related domains, making it ideal for evaluating the performance of domain adaptation methods across diverse visual domains. DomainNet, on the other hand, is a more challenging dataset, comprising a large number of categories across six different domains, including real-world images, clipart, and sketches, among others. Both datasets offer unique challenges in terms of domain shift and data distribution, making them well-suited for testing the generalization ability of domain adaptation models.

(A) *OfficeHome*

**Figure 4:**
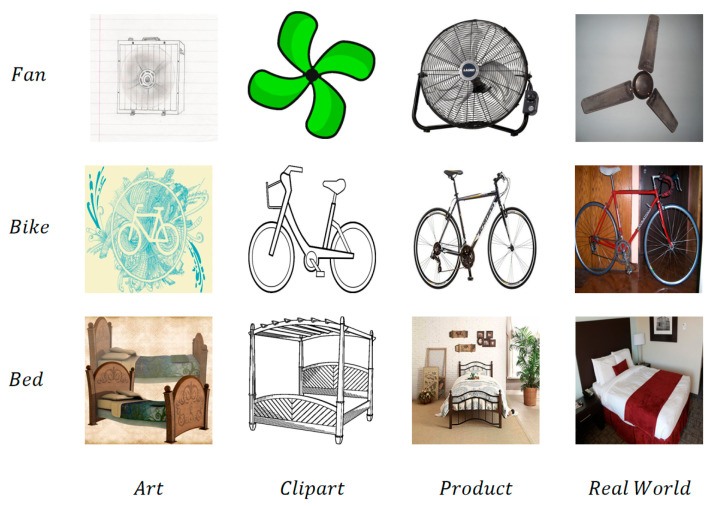
OfficeHome dataset example.

The OfficeHome dataset is a widely used benchmark in the field of domain adaptation, consisting of images from four distinct domains: Art, Clipart, Product, and Real World. Each domain contains images from 65 object categories, with a total of 15,500 images. The diversity of the domains makes it particularly suitable for evaluating algorithms in terms of their ability to handle different types of visual data, ranging from digital artworks to real-world photos. This dataset is specifically designed to assess the effectiveness of domain adaptation methods in addressing the challenge of domain shift, as the images across these domains exhibit significant differences in style, background, and content. The complexity of this dataset has made it a popular choice for testing models that aim to generalize across various domains while maintaining high performance. An example of its dataset is shown in Figure 4.

(B) *DomainNet*

**Figure 5:**
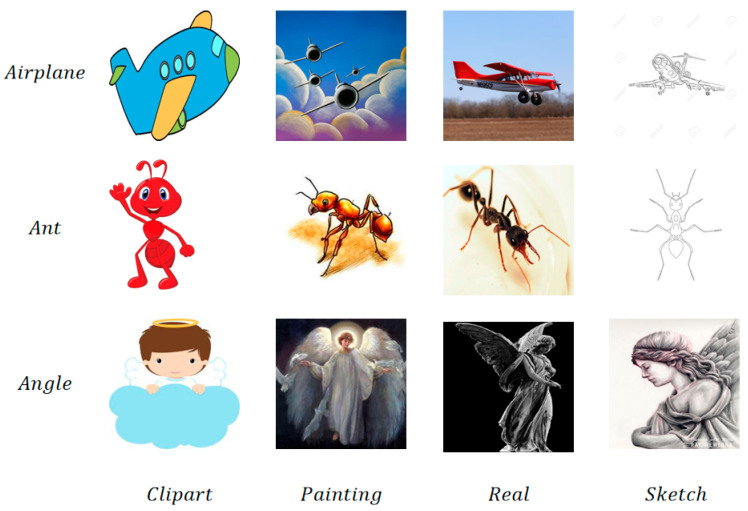
DomainNet dataset example image.

DomainNet is a large-scale dataset designed to evaluate domain adaptation techniques, comprising over 600,000 images spread across six distinct domains: Real, Clipart, Infograph, Painting, QuickDraw, and Sketch. These domains cover a wide range of visual data types, from realistic photos to hand-drawn sketches and digital drawings, providing a diverse set of challenges for domain adaptation algorithms. DomainNet contains 345 object categories, making it one of the most comprehensive datasets for testing models in the context of cross-domain generalization. The significant variations in image style, content, and visual representation between these domains present a formidable challenge for domain adaptation, making DomainNet an ideal benchmark for assessing the robustness and versatility of domain adaptation methods. An example of its dataset is shown in Figure 5.

### 4.2. Evaluation Metrics

In this study, the performance of the domain adaptation model is evaluated using accuracy (ACC) as the primary metric. Accuracy measures the proportion of correctly classified samples in the target domain, reflecting the model’s ability to generalize to the target domain after adapting from the source domain. Specifically, accuracy is calculated by comparing the predicted labels with the true labels in the target domain. The accuracy is formulated as follows:

(33) $$Acc=\frac{1}{N_{T}}\sum_{i=1}^{N_{T}}I(y_{i}^{T}={y\prime }_{i}^{T})$$

where $N_{T}$ is the number of samples in the target domain, $y_{i}^{T}$ is the true label of the i-th sample in the target domain, ${y\prime }_{i}^{T}$ is the predicted label, and I is the indicator function that equals 1 if the prediction is correct $\left(y_{i}^{T}={y\prime }_{i}^{T}\right)$, and 0 otherwise.

## 5. Experiment

To comprehensively evaluate the proposed unsupervised domain adaptation method based on relative entropy regularization and measure propagation, a series of experiments are designed in this chapter. These include comparison experiments to validate the model’s performance on the target domain, hyperparameter sensitivity experiments to analyze the impact of key parameters on the results, ablation experiments to explore the role of each component, and visualization experiments to visually demonstrate the feature distribution and distribution alignment effects. These experiments are conducted on multiple benchmark datasets, aiming to systematically verify the method’s effectiveness, robustness, and theoretical advantages.

### 5.1. Experimental Details

In the experiments, the model is trained for 300 epochs using a ResNet50 pre-trained on ImageNet as the backbone to extract deep features. The Adam optimizer is used with an initial learning rate of 0.001, which is dynamically adjusted via cosine annealing. The batch size is set to 32, and all experiments are conducted on a computing platform equipped with an NVIDIA 4090D GPU to ensure computational efficiency and consistency. The model weights are fine-tuned through random initialization, and hyperparameters, such as the relative entropy regularization weight and measure propagation weight, are optimized on the validation set to ensure the fairness and reproducibility of the experimental results.

### 5.2. Performance Comparison Experiment

The training indicator evaluation of the comparative experiment follows the unsupervised domain adaptation task. For the OfficeHome dataset, its four domains C, P, R, and A and the other three domains are tested for domain adaptation. Each result is tested three times with different random seeds and the average is taken. The average accuracy of 12 experiments is used to express the overall evaluation of the dataset.

The training DomainNet dataset contains six data domains. Given that the Infograph and QuickDraw domains are too different from other data, and it is difficult to obtain better feature expression when using Resnet50 as the backbone, only four of the six domains in the dataset (Real, Painting, Sketch, Clipart) are selected for the same test as OfficeHome. Its overall evaluation indicator is still the average accuracy of the 12 pairs of tasks. The experimental results are shown in Table 1.

The experimental results demonstrate that our proposed unsupervised domain adaptation method based on relative entropy regularization and measure propagation achieves an average accuracy of 72.3% on the OfficeHome dataset, outperforming all baseline models, including ToAlign (71.8%). This result confirms the overall effectiveness of our method in enhancing cross-domain generalization.

From an information-theoretic standpoint, relative entropy regularization effectively constrains the predicted label distribution of the target domain to align with a reference distribution derived from the source domain via KL divergence. This helps reduce prediction uncertainty in the target domain, especially in domain pairs where category-level alignment is critical (e.g., A2P and C2P). On the other hand, measure propagation promotes global consistency in feature representation by transferring probability mass from the source to the target domain, which is particularly beneficial in domain pairs such as R2P and P2R, where structural differences in the feature space are more pronounced.

Notably, our method excels on several domain pairs with relatively moderate distribution shifts, including A2P (77.5%), C2P (76.5%), and R2P (85.2%). These results suggest that our method effectively captures both local label consistency and global feature structure in such settings. However, it does not achieve the best results on more challenging pairs like A2C (59.0%), C2A (66.5%), and P2C (57.5%), where the domain shift is more complex and localized. In these cases, the current formulation of KL divergence and the pseudo-measure approximation may lack the granularity needed for fine-grained adaptation, limiting performance.

Despite these limitations, the joint optimization of relative entropy regularization and measure propagation provides a strong balance between alignment accuracy and structural generalization. This synergy results in consistently competitive performance across a wide range of domain pairs, validating the robustness and theoretical soundness of the proposed method.

**Figure 6:**
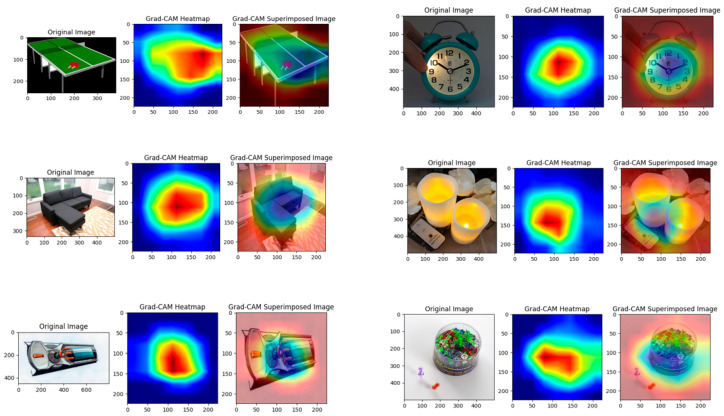
The result of the Grad-CAM algorithm in the target domain of the OfficeHome dataset.

This paper also gives the Grad-CAM image of the OfficeHome dataset, as shown in Figure 6.

The Grad-CAM [40,41] visualization results show the performance of our proposed unsupervised domain adaptation method based on relative entropy regularization and measure propagation on the OfficeHome target domain, for six examples. Each example includes the original image, Grad-CAM heat map, and superimposed image. The heat map focuses on the key areas of the target object, and the superimposed image clearly shows the target part that the model focuses on, indicating that relative entropy regularization constrains the consistency of the target domain distribution with the source domain through KL divergence, and measure propagation enhances the distinguishing ability of features through probability measure alignment, thereby achieving accurate category recognition in the target domain, verifying the effectiveness of the method. This paper also gives the experimental results of other datasets, as shown in Table 2.

The experimental results show that our proposed unsupervised domain adaptation method based on relative entropy regularization and measure propagation achieves an average performance of 46.2% on the DomainNet dataset, significantly outperforming other baseline models (such as ToAlign with 45.4%). This verifies the effectiveness of our method in handling large-scale, cross-domain tasks with strong distribution heterogeneity. The complexity of DomainNet, with larger distributional differences between domains, imposes higher demands on information-theoretic methods. Relative entropy regularization precisely constrains the consistency between the predicted distribution of the target domain and the reference distribution of the source domain using KL divergence, reducing information loss. Measure propagation, by propagating the source domain’s probability measures to generate pseudo-measures for the target domain, enhances the global alignment of the feature space. The method performs exceptionally well on challenging domain pairs such as A2C (51.2%) and P2A (57.7%), contributing to the overall improvement in average performance.

Similarly, our method does not perform the best on all domain pairs in the DomainNet dataset. For example, it is does not perform as well as some models (such as PAN and FixBi) on A2P (49.6%), C2P (34.2%), and P2R (36.4%). This may be due to the larger distribution heterogeneity and noise in the DomainNet dataset, where relative entropy regularization and measure propagation may require stronger local adaptation capabilities in some extreme scenarios. Nevertheless, the overall robustness of the method benefits from the information-theory-driven joint optimization. The use of KL divergence to quantify distributional differences and measure propagation to model global structure allows the method to maintain significant performance advantages on large-scale, complex datasets, highlighting the applicability and scalability of the theoretical design.

### 5.3. Ablation Experiment

To comprehensively evaluate the contribution of each component in our proposed unsupervised domain adaptation method, based on relative entropy regularization and measure propagation, this section presents ablation experiments on the OfficeHome dataset. The focus is on analyzing the performance of four representative domain pairs: A2P, C2P, P2A, and R2C. By systematically removing key modules (such as relative entropy regularization or measure propagation), we verify the contribution of each part to the overall model performance. The experimental results are shown in Table 3.

The ablation experiment results show that our proposed unsupervised domain adaptation method, based on relative entropy regularization and measure propagation, performs excellently on the A2P, C2P, P2A, and R2C domain pairs of the OfficeHome dataset. The complete model (ours) achieves the best performance across all domain pairs, with A2P reaching 77.5%, C2P 76.5%, P2A 68.0%, and R2C 61.8%. After removing relative entropy regularization, A2P drops to 75.2%, C2P to 74.3%, P2A to 65.7%, and R2C to 59.4%. This indicates that KL divergence plays a critical role in constraining the consistency between the target domain’s predicted distribution and the source domain’s reference distribution. The impact is especially noticeable in A2P and C2P, where there are large distributional differences, validating the necessity of information-theoretic alignment.

After removing measure propagation, A2P drops to 76.0%, C2P to 75.1%, P2A to 66.5%, and R2C to 60.3%. This shows that measure propagation, by propagating the source domain’s probability measures to generate pseudo-measures for the target domain, makes a significant contribution to the global alignment of the feature space, especially in domain pairs with higher heterogeneity, such as P2A and R2C. Although performance decreases when either of the two modules is removed, the collaborative effect of the complete model significantly improves performance, fully demonstrating the complementary advantages of relative entropy regularization and measure propagation in information-theory-driven domain adaptation.

**Figure 7:**
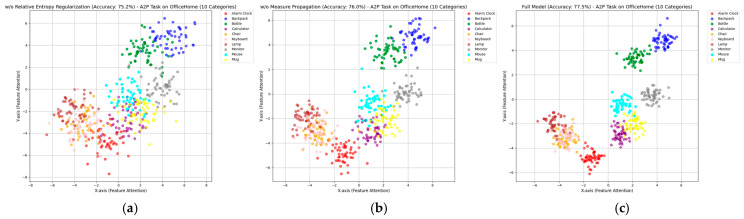
T-SNE graphs under different ablation conditions. (**a**) w/o Relative Entropy Regularization, (**b**) w/o Measure Propagation, (**c**) Ours (Full Model).

Secondly, for the P2A task, this paper selected 10 categories, with 50 samples in each category, and used T-SNE [46] to visualize the results of three different ablation experiments. The experimental results are shown in Figure 7.

The T-SNE visualization in the experimental results shows the feature distribution of our proposed unsupervised domain adaptation method, based on relative entropy regularization and measure propagation, on the A2P task of the OfficeHome dataset, involving 10 categories (from Alarm Clock to Mug). The complete model (ours, with an accuracy of 77.5%) exhibits the clearest category clustering in its feature distribution (Figure 7c), with compact clusters and clear boundaries for the 10 categories. This indicates that relative entropy regularization constrains the consistency between the target domain and the source domain distributions using KL divergence, while measure propagation achieves global feature space alignment by propagating probability measures, significantly enhancing the separability between categories and reflecting the method’s optimal performance.

After removing relative entropy regularization (Figure 7a, accuracy 75.2%) or measure propagation (Figure 7b, accuracy 76.0%), the clustering effect of the feature distribution significantly worsens. In Figure 7a, the category clusters become more scattered and partially overlap, indicating that the absence of the KL divergence constraint increases the deviation between the target domain’s predicted distribution and the source domain’s reference distribution, exacerbating information loss. In Figure 7b, although the category distribution is more concentrated compared to Figure 7a, it is still more dispersed than in the complete model, indicating the indispensable role of measure propagation in global feature alignment. The performance degradation in these two figures validates the complementary role of relative entropy regularization and measure propagation in information-theory-driven domain adaptation.

### 5.4. Hyperparameter Sensitivity Experiments

In order to deeply evaluate the robustness of our unsupervised domain adaptation method based on relative entropy regularization and measure propagation, this section conducts hyperparameter sensitivity experiments on the OfficeHome dataset, focusing on analyzing the impact of key hyperparameters on model performance. First, this paper experiments on conventional hyperparameters, such as the optimizer. Secondly, we pay special attention to changes in relative entropy regularization weight $\lambda_{re}$ and measure propagation weight $\lambda_{mp}$. By testing different value ranges on the four representative domain pairs of A2C, C2A, P2R, and R2P, we systematically explore the impact of hyperparameter settings on accuracy, thereby ensuring the stability and optimal performance of the method in practical applications. The experimental results are shown in Table 4.

The results of hyperparameter sensitivity experiments show that our proposed unsupervised domain adaptation method, based on relative entropy regularization and measure propagation, has the best performance on the A2C, C2A, P2R, and R2P domain pairs of the OfficeHome dataset, with the Adam optimizer reaching 59.0%, 66.5%, 82.2%, and 85.2%, respectively, verifying the high efficiency of Adam in complex optimization that combines KL divergence constraints and probabilistic measure propagation. In contrast, RMSprop performs slightly worse on A2C (56.7%), C2A (64.2%), P2R (80.4%), and R2P (83.6%), indicating that it converges slowly when dealing with information-theoretic-driven distribution alignment; the performance of Adagrad and SGD is between the two, with Adagrad slightly outperforming SGD (A2C 55.9%, C2A 65.0%, P2R 79.8%) on A2C (57.3%), C2A (63.8%), and P2R (81.0%), but still lower than Adam overall, reflecting the differences between different optimizers in dynamic learning rate adjustment and non-convex optimization.

**Figure 8:**
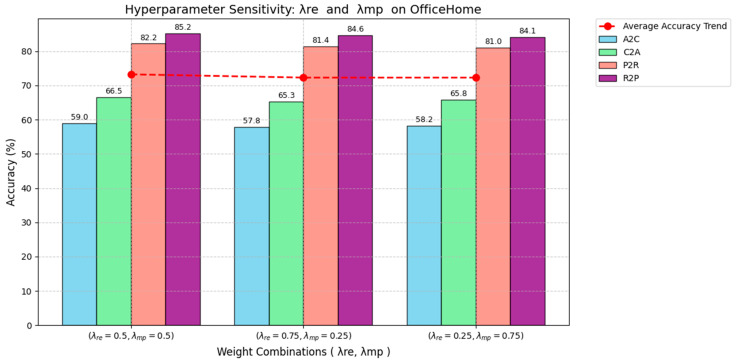
Hyperparameter sensitivity experiments of $\lambda_{re}$ and $\lambda_{mp}$.

These results further illustrate that the Adam optimizer can better adapt to the complex requirements of relative entropy regularization to minimize distributional differences through KL divergence and measure propagation to align feature distributions through probability measures, especially in domain pairs with high distribution heterogeneity, such as A2C and R2P. The performance degradation of RMSprop, Adagrad, and SGD indicates that they may be limited by the dynamic adjustment of learning rate or convergence efficiency in information-theory-driven joint optimization, which verifies the robustness and applicability of Adam as the default optimizer and provides an important reference for performance optimization of models on different domain pairs. In order to intuitively display the experimental results, an image of the hyperparameter sensitivity experiment is given, as shown in Figure 8.

The results of hyperparameter sensitivity experiments show that the unsupervised domain adaptation method based on relative entropy regularization and measure propagation proposed by us performs best on the A2C, C2A, P2R, and R2P domain pairs of the OfficeHome dataset when the relative entropy weight is $\lambda_{re}$ = 0.5 and the measure propagation weight is $\lambda_{mp}$ = 0.5, reaching 59.0%, 66.5%, 82.2%, and 85.2%, respectively, verifying the synergistic optimization effect of weight balance on the KL divergence constraint and probability measure alignment; when $\lambda_{re}$ = 0.75 and $\lambda_{mp}$ = 0.25, or $\lambda_{re}$ = 0.25 and $\lambda_{mp}$ = 0.75, the performance decreases slightly (for example, A2C drops to 57.8% and 58.2%, respectively), indicating that weight imbalance may weaken the distribution alignment ability driven by information theory, especially on domain pairs with high distribution heterogeneity, such as A2C and C2A.

### 5.5. Performance on Text Data for Sentiment Classification

In order to further verify the generalization ability of our proposed unsupervised domain adaptation method based on relative entropy regularization and measure propagation on different types of data, we designed an experiment for text data, focusing on the cross-domain sentiment classification task. Sentiment classification is an important natural language processing task widely used in social media analysis, user comment mining, and other fields. However, due to differences in the distribution of language styles and expressions in different text domains, directly applying the model trained in the source domain to the target domain usually leads to performance degradation. Therefore, this experiment aims to verify the effectiveness of this method in text domain adaptation.

We selected the Amazon Reviews Dataset as the experimental dataset, which is a benchmark dataset for cross-domain sentiment classification. It contains user reviews of product categories such as Books (source domain, 10,000 labeled reviews, 5000 positive and 5000 negative) and Electronics (target domain, 10,000 unlabeled reviews), reflecting the changes in language style and vocabulary distribution due to category differences. In the experiment, we used the pre-trained BERT-base-uncased model as the feature extractor, fine-tuned it with Books domain data, and added a fully connected classifier on BERT, combined with relative entropy regularization and measure propagation. The experiment was run on the NVIDIA 4090D GPU to ensure efficiency. The experimental results are shown in Table 5.

Table 5 shows the experimental results of sentiment classification of our unsupervised domain adaptation method on the Amazon Reviews dataset (Books → Electronics), where our method achieved an accuracy of 78.4%, significantly better than the baseline model: BERT (Source Only) only achieved an accuracy of 65.2%, indicating that the performance is poor without domain adaptation; DANN and CDAN achieved 71.3% and 73.5%, respectively, showing that adversarial training can alleviate distributional differences, but the effect is limited; ToAlign reached 76.1%, which is close to our results but still inferior. This shows that relative entropy regularization and measure propagation effectively improve the performance of cross-domain sentiment classification by constraining distribution consistency and global semantic alignment through KL divergence, especially in text tasks with large differences in language style, verifying the robustness and superiority of the method on text data.

## 6. Conclusions

This paper proposes an unsupervised domain adaptation method based on relative entropy regularization and measure propagation. By incorporating KL divergence as an information-theoretic constraint and propagating probability measures for structural alignment, the method significantly improves generalization on target domains. Experimental results on the OfficeHome and DomainNet datasets demonstrate strong performance, with average accuracies of 72.3% and 46.2%, respectively, outperforming several competitive baselines. The proposed framework offers a principled and scalable solution for reducing distribution discrepancies and enhancing model robustness under moderate domain shifts.

While accuracy is a widely used evaluation metric and effectively demonstrates the performance of our approach, we acknowledge its limitations in capturing model behavior under class imbalance or skewed label distributions—common issues in real-world domain adaptation tasks. Relying solely on accuracy may overlook cases where the model performs poorly on minority classes. In future work, we plan to incorporate additional evaluation metrics such as F1-score or balanced accuracy and perform stratified analysis to better understand model behavior across different categories.

Despite its advantages, the proposed method still exhibits limitations when facing highly heterogeneous domain pairs, where global alignment alone may be insufficient. To address this, future research will focus on integrating local structure-aware adaptation strategies and enhancing the flexibility of pseudo-measure modeling to handle fine-grained distribution shifts. Additionally, scaling the framework to large-scale datasets and real-time applications remains an important challenge. Potential deployment scenarios include cross-institutional medical image analysis, multi-sensor perception in autonomous driving, and cross-platform sentiment classification, where distributional gaps and label scarcity are prevalent. These future directions will further improve the adaptability, interpretability, and real-world applicability of our method in complex cross-domain scenarios.

## Figures and Tables

**Table 1:** Experimental results on an OfficeHome dataset.

Model	A2C	A2P	A2R	C2A	C2P	C2R	P2A	P2C	P2R	R2A	R2C	R2P	Avg
ResNet50 [26]	34.9	50.0	58.0	37.4	41.9	46.2	38.5	31.2	60.4	53.9	41.2	59.9	46.1
MCD [27]	48.9	68.3	74.6	61.3	67.6	68.8	57.0	47.1	75.1	69.1	52.2	79.6	64.1
CDANs [28]	50.7	70.6	76.0	57.6	70.0	70.0	57.4	50.9	77.3	70.9	56.7	81.6	65.8
ALDA [29]	53.7	70.1	76.4	60.2	72.6	71.5	56.8	51.9	77.1	70.2	56.3	82.1	66.6
TADA [30]	53.1	72.3	77.2	59.1	71.2	72.1	59.7	53.1	78.4	72.4	60.0	82.9	67.6
MCD [31]	54.9	73.7	77.8	60.0	71.4	71.8	61.2	53.6	78.1	72.5	60.2	82.3	68.1
BNM	56.2	73.7	79.0	63.1	73.6	74.0	62.4	54.8	80.7	72.4	58.9	83.5	69.4
GSDA [32]	61.3	76.1	79.4	65.4	73.3	74.3	65.0	53.2	80.0	72.2	60.6	83.1	70.3
GVB [33]	57.0	74.7	79.8	64.6	74.1	74.6	65.2	55.1	81.0	74.6	59.7	84.3	70.4
E-Mix [34]	57.7	76.0	79.8	63.6	74.1	75.0	63.4	56.4	79.7	72.8	**62.4**	**85.5**	70.6
HDA [35]	56.8	75.2	79.8	65.1	73.9	75.2	66.3	56.7	81.8	75.4	59.7	84.7	70.9
Meta-Align [36]	**59.3**	76.0	80.2	65.7	74.7	75.1	65.7	56.5	81.6	74.1	61.1	85.2	71.3
ToAlign [37]	57.9	**76.9**	80.8	**66.7**	75.6	77.0	67.8	57.0	**82.5**	**75.1**	60.0	84.9	71.8
Norm-AE-SPL [38]	51.6	76.0	80.6	63.0	**77.0**	**78.4**	62.9	50.7	81.2	66.3	52.8	82.9	68.6
SACAEM [39]	55.2	70.7	75.7	60.7	68.3	68.9	62.3	55.9	78.5	72.8	82.1	82.8	69.5
Ours	59.0	**77.5**	**81.0**	66.5	76.5	77.2	**68.0**	**57.5**	82.2	75.0	61.8	85.2	**72.3**

The best result is in bold.

**Table 2:** Experimental results on a DomainNet dataset.

Model	R2C	R2P	R2S	C2R	C2P	C2S	P2R	P2C	P2S	S2R	S2C	S2P	Avg
ResNet50	41.6	42.7	29.6	42.4	27.2	32.1	49.5	32.5	26.7	38.7	40.8	27.5	35.9
MSTN [40]	27.2	32.9	24.3	28.1	21.1	24.1	30.7	19.8	22.5	24.3	26.2	23.5	25.4
RSDA [41]	27.2	35.8	24.3	36.9	24.9	31.1	41.3	26.1	24.7	29.4	26.2	27.7	29.6
MCD	36.3	36.5	24.9	40.3	25.8	32.1	43.6	29.6	25.7	34.1	39.1	26.8	32.9
DANN	45.9	44.5	35.4	46.8	30.5	36.7	48.0	34.7	32.1	47.1	46.4	38.4	40.5
CAN [42]	40.7	37.7	33.7	**54.9**	31.4	37.3	51.0	33.6	30.9	**52.1**	42.1	32.0	39.8
PAN [43]	49.2	48.1	36.4	49.6	33.2	38.7	51.8	36.0	32.9	49.1	50.9	39.8	43.0
CDANs	50.1	48.3	39.0	50.0	33.3	39.3	52.2	36.4	33.6	48.4	49.2	38.6	43.2
HDA	46.3	47.5	34.3	49.9	33.9	37.9	55.2	40.8	32.7	49.0	49.7	40.0	43.1
FixBi	51.1	49.1	**39.6**	50.0	**34.5**	41.1	52.2	36.4	33.6	50.8	53.5	41.6	44.5
ToAlign	50.8	**50.7**	35.1	49.5	33.8	41.4	**57.9**	**43.5**	36.2	47.9	**55.5**	41.6	45.4
D^3^GU [44]	51.0	50.3	35.6	49.8	34.0	41.5	57.5	43.1	36.3	48.1	55.3	41.6	45.7
DCST [45]	51.1	50.1	35.9	50.0	34.1	41.6	57.5	43.1	**36.5**	48.5	55.0	41.3	45.3
Ours	**51.2**	49.6	36.1	50.3	34.2	**41.7**	57.7	43.3	**36.4**	48.4	55.2	**41.7**	**46.2**

The best result is in bold.

**Table 3:** Ablation experiment (OfficeHome dataset).

Model	A2P	C2P	P2A	R2C
Ours (Full Model)	77.5	76.5	68.0	61.8
w/o Relative Entropy Regularization	75.2	74.3	65.7	59.4
w/o Measure Propagation	76.0	75.1	66.5	60.3

**Table 4:** Hyperparameter sensitivity experiments (OfficeHome dataset).

Optimizer	A2C	C2A	P2R	R2P
RMSprop	56.7	64.2	80.4	83.6
Adagrad	57.3	63.8	81.0	84.1
SGD	55.9	65.0	79.8	83.2
Adam	59.0	66.5	82.2	85.2

**Table 5:** Experimental results on text sentiment classification.

Method	ACC
BERT (Source Only)	65.2%
DANN	71.3%
CDAN	73.5%
ToAlign	76.1%
**Ours**	**78.4%**

## Data Availability

The original contributions presented in this study are included in the article. Further inquiries can be directed to the corresponding author(s).

## References

[1] Liu X., Yoo C., Xing F., Oh H., El Fakhri G., Kang J.W., Woo J. (2022). Deep unsupervised domain adaptation: A review of recent advances and perspectives. APSIPA Trans. Signal Inf. Process..

[2] Park G.Y., Lee S.W. Information-theoretic regularization for multi-source domain adaptation. Proceedings of the IEEE/CVF International Conference on Computer Vision.

[3] Deng W., Zhao L., Liao Q., Guo D., Kuang G., Hu D., Pietikainen M., Liu L. (2021). Informative feature disentanglement for unsupervised domain adaptation. IEEE Trans. Multimed..

[4] Ahmed S.M., Raychaudhuri D.S., Paul S., Oymak S., Roy-Chowdhury A.K. Unsupervised multi-source domain adaptation without access to source data. Proceedings of the IEEE/CVF Conference on Computer Vision and Pattern Recognition.

[5] Guan D., Huang J., Xiao A., Lu S., Cao Y. (2021). Uncertainty-aware unsupervised domain adaptation in object detection. IEEE Trans. Multimed..

[6] Fang Z., Lu J., Liu F., Zhang G. (2022). Semi-supervised heterogeneous domain adaptation: Theory and algorithms. IEEE Trans. Pattern Anal. Mach. Intell..

[7] Ma A., Li J., Lu K., Zhu L., Shen H.T. (2021). Adversarial entropy optimization for unsupervised domain adaptation. IEEE Trans. Neural Netw. Learn. Syst..

[8] Shi Y., Ying X., Yang J. (2022). Deep unsupervised domain adaptation with time series sensor data: A survey. Sensors.

[9] Oza P., Sindagi V.A., Vs V., Patel V.M., Sharmini V.V. (2023). Unsupervised domain adaptation of object detectors: A survey. IEEE Trans. Pattern Anal. Mach. Intell..

[10] Xu T., Chen W., Wang P., Wang F., Li H., Jin R. (2021). Cdtrans: Cross-domain transformer for unsupervised domain adaptation. arXiv.

[11] Mirza M.J., Micorek J., Possegger H., Bischof H. The norm must go on: Dynamic unsupervised domain adaptation by normalization. Proceedings of the IEEE/CVF Conference on Computer Vision and Pattern Recognition.

[12] Huang J., Guan D., Xiao A., Lu S., Shao L. Category contrast for unsupervised domain adaptation in visual tasks. Proceedings of the IEEE/CVF Conference on Computer Vision and Pattern Recognition.

[13] Yu W., Sohrabi F., Jiang T. (2022). Role of deep learning in wireless communications. IEEE BITS Inf. Theory Mag..

[14] Ali A., Anam S., Ahmed M.M. (2023). Shannon entropy in artificial intelligence and its applications based on information theory. J. Appl. Emerg. Sci..

[15] Chung W., Zhang Y., Pan J. (2023). A theory-based deep-learning approach to detecting disinformation in financial social media. Inf. Syst. Front..

[16] Walunj V., Gharibi G., Alanazi R., Lee Y. (2022). Defect prediction using deep learning with Network Portrait Divergence for software evolution. Empir. Softw. Eng..

[17] Cui S., Wang S., Zhuo J., Li L., Huang Q., Tian Q. (2021). Fast batch nuclear-norm maximization and minimization for robust domain adaptation. arXiv.

[18] Hamidi S.M. (2024). The Interplay of Information Theory and Deep Learning: Frameworks to Improve Deep Learning Efficiency and Accuracy.

[19] Shwartz Ziv R., LeCun Y. (2024). To compress or not to compress—Self-supervised learning and information theory: A review. Entropy.

[20] Wu C., Zhang J. (2022). Robust semi-supervised spatial picture fuzzy clustering with local membership and KL-divergence for image segmentation. Int. J. Mach. Learn. Cybern..

[21] Sanokowski S., Hochreiter S., Lehner S. (2024). A diffusion model framework for unsupervised neural combinatorial optimization. arXiv.

[22] Lee K.S., Tran N.T., Cheung N.M. Infomax-gan: Improved adversarial image generation via information maximization and contrastive learning. Proceedings of the IEEE/CVF Winter Conference on Applications of Computer Vision.

[23] Li J., Ren Y., Deng K. FairGAN: GANs-based fairness-aware learning for recommendations with implicit feedback. Proceedings of the ACM Web Conference 2022.

[24] Ge P., Ren C.X., Xu X.L., Yan H. (2023). Unsupervised domain adaptation via deep conditional adaptation network. Pattern Recognit..

[25] Zhang Y., Wang Z., He W. Class relationship embedded learning for source-free unsupervised domain adaptation. Proceedings of the IEEE/CVF Conference on Computer Vision and Pattern Recognition.

[26] He K., Zhang X., Ren S., Sun J. Deep residual learning for image recognition. Proceedings of the IEEE Conference on Computer Vision and Pattern Recognition.

[27] Saito K., Watanabe K., Ushiku Y., Harada T. Maximum classifier discrepancy for unsupervised domain adaptation. Proceedings of the IEEE Conference on Computer Vision and Pattern Recognition.

[28] Long M., Cao Z., Wang J., Jordan M.I. (2018). Conditional adversarial domain adaptation. Adv. Neural Inf. Process. Syst..

[29] Chen M., Zhao S., Liu H., Cai D. (2020). Adversarial-learned loss for domain adaptation. Proc. AAAI Conf. Artif. Intell..

[30] Hung C.C., Lange L., Strötgen J. (2023). TADA: Efficient task-agnostic domain adaptation for transformers. arXiv.

[31] Zhang Y., Liu T., Long M., Jordan M. Bridging theory and algorithm for domain adaptation. Proceedings of the 36th International Conference on Machine Learning.

[32] Hu L., Kan M., Shan S., Chen X. Unsupervised domain adaptation with hierarchical gradient synchronization. Proceedings of the IEEE/CVF Conference on Computer Vision and Pattern Recognition.

[33] Cui S., Wang S., Zhuo J., Su C., Huang Q., Tian Q. Gradually vanishing bridge for adversarial domain adaptation. Proceedings of the IEEE/CVF Conference on Computer Vision and Pattern Recognition (CVPR).

[34] Zhong L., Fang Z., Liu F., Lu J., Yuan B., Zhang G. (2021). How does the combined risk affect the performance of unsupervised domain adaptation approaches?. Proc. AAAI Conf. Artif. Intell..

[35] Cui S., Jin X., Wang S., He Y., Huang Q. (2020). Heuristic Domain Adaptation.

[36] Wei G., Lan C., Zeng W., Chen Z. Metaalign: Coordinating domain alignment and classification for unsupervised domain adaptation. Proceedings of the IEEE/CVF Conference on Computer Vision and Pattern Recognition.

[37] Wei G., Lan C., Zeng W., Zhang Z., Chen Z. (2021). Toalign: Task-oriented alignment for unsupervised domain adaptation. Adv. Neural Inf. Process. Syst..

[38] Wang Q., Meng F., Breckon T.P. (2023). Data augmentation with norm-AE and selective pseudo-labelling for unsupervised domain adaptation. Neural Netw..

[39] Li Z., Wang B., Chen Y. (2024). A contrastive deep learning approach to cryptocurrency portfolio with us treasuries. J. Comput. Technol. Appl. Math..

[40] Chang W.G., You T., Seo S., Kwak S., Han B. Domain-specific batch normalization for unsupervised domain adaptation. Proceedings of the IEEE/CVF Conference on Computer Vision and Pattern Recognition.

[41] Deng W., Liao Q., Zhao L., Guo D., Kuang G., Hu D., Liu L. (2021). Joint clustering and discriminative feature alignment for unsupervised domain adaptation. IEEE Trans. Image Process..

[42] Lee S., Kim D., Kim N., Jeong S.G. Drop to adapt: Learning discriminative features for unsupervised domain adaptation. Proceedings of the IEEE/CVF International Conference on Computer Vision.

[43] Cao Z., Ma L., Long M., Wang J. Partial adversarial domain adaptation. Proceedings of the European Conference on Computer Vision (ECCV).

[44] Zhang L., Xu L., Motamed S., Chakraborty S., De la Torre F. D3GU: Multi-target Active Domain Adaptation via Enhancing Domain Alignment. Proceedings of the IEEE/CVF Winter Conference on Applications of Computer Vision.

[45] Cheng Y., Yao P., Xu L., Chen M., Liu P., Shao P., Shen S., Xu R.X. (2025). DCST: Dual Cross-Supervision for Transformer-based Unsupervised Domain Adaptation. Neural Netw..

[46] Van der Maaten L., Hinton G. (2008). Visualizing data using t-SNE. J. Mach. Learn. Res..

